# Altmetrics Attention Scores for Randomized Controlled Trials in Total Joint Arthroplasty Are Reflective of High Scientific Quality: An Altmetrics-Based Methodological Quality and Bias Analysis

**DOI:** 10.5435/JAAOSGlobal-D-20-00187

**Published:** 2020-12-02

**Authors:** Kyle N. Kunze, Michelle Richardson, David N. Bernstein, Ajay Premkumar, Nicolas S. Piuzzi, Alexander S. McLawhorn

**Affiliations:** From the Department of Orthopaedic Surgery, Hospital for Special Surgery, New York (Dr. Kunze, Dr. Premkumar, Dr. McLawhorn); University of Rochester School of Medicine, Rochester, NY (Ms. Richardson); the Department of Orthopaedic Surgery, Massachusetts General Hospital, Boston, MA (Dr. Bernstein); and the Department of Orthopaedic Surgery, Cleveland Clinic Foundation, Cleveland, OH (Dr. Piuzzi).

## Abstract

The Altmetric Attention Score (AAS) has been associated with citation rates across medical and surgical disciplines. However, factors that drive high AAS remain poorly understood and there remains multiple pitfalls to correlating these metrics alone with the quality of a study. The purpose of the current study was to determine the relationship between methodologic and study biases and the AAS in randomized controlled trials (RCTs) published in total joint arthroplasty journals.

**Methods:**

All RCTs from 2016 published in *The Journal of Arthroplasty*, *The Bone and Joint Journal*, *The Journal of Bone and Joint Surgery*, *Clinical Orthopedics and Related Research*, *The Journal of Knee Surgery*, *Hip International*, and *Acta Orthopaedica* were extracted. Methodologic bias was graded with the JADAD scale, whereas study bias was graded with the Cochrane risk of bias tool for RCTs. Publication characteristics, social media attention (Facebook, Twitter, and Mendeley), AAS, citation rates, and bias were analyzed.

**Results:**

A total of 42 articles were identified. The mean (±SD) citations and AAS per RCT was 17.8 ± 16.5 (range, 0 to 78) and 8.0 ± 15.4 (range, 0 to 64), respectively. The mean JADAD score was 2.6 ± 0.94. No statistically significant differences were observed in the JADAD score or total number of study biases when compared across the seven journals (*P* = 0.57 and *P* = 0.27). Higher JADAD scores were significantly associated with higher AAS scores (β = 6.7, *P* = 0.006) but not citation rate (*P* = 0.16). The mean number of study biases was 2.0 ± 0.93 (range, 0 to 4). A greater total number of study biases was significantly with higher AAS scores (β = −8.0, *P* < 0.001) but not citation rate (*P* = 0.10). The AAS was a significant and positive predictor of citation rate (β = 0.43, *P* = 0.019).

**Conclusion:**

High methodologic quality and limited study bias markedly contribute to the AAS of RCTs in the total joint arthroplasty literature. The AAS may be used as a proxy measure of scientific quality for RCTs, although readers should still critically appraise these articles before making changes to clinical practice.

The impact of research has traditionally been measured by citation rate.^1^ Although a commonly used metric, article citation rate has inherent limitations such as failing to take into account scholarly impact transmitted through other outlets such as social media platforms and also may require years of citation accrual until the impact of an article is apparent.^2,3^ Furthermore, citation rates do not necessarily correlate with quality. Given these limitations, Altmetric, a data science company, developed the Altmetric Attention Score (AAS) that can track and quantify the social media attention an individual article receives and subsequently its impact in real-time.^4-6^ These benefits have led to a large increase in the number of studies investigating the AAS and its relation to citation rate and impact across multiple fields ranging from cardiology^7^ and neurology^8^ to orthopaedic surgery.^9,10^ In fact, many journals in orthopaedic surgery now display the AAS “donut” on each article's page such that it is easily accessible. Given the increasing visibility of Altmetrics and its role in understanding research in an age where social media platforms have become mediums through which research is disseminated, there is a need to better understand the factors that drive AAS.

Previous studies have focused on evaluating the relationships between AAS and citation rate, as well as article characteristics that are associated with higher AAS.^9^ Although defining these relationships are important, the influence of external factors that may be associated with higher AAS, such as the methodologic quality or number of biases in a study, is less understood. The audience reached by social media, whether consisting of individuals in academics or the general public, may disproportionately increase the AAS of articles that discuss trending topics without regard for their methodology. In an analysis of dementia biomarker studies, MacKinnon et al^11^ determined that neither the impact factor nor the citation rate was associated with methodologic quality, whereas this group was unable to analyze AAS because of incomplete data. Nonetheless, these relationships may vary by field and the tools used to determine methodological quality. For example, the JADAD scale that is frequently used in the orthopaedic surgery literature to appraise randomized controlled trials (RCTs) may assess components of methodologic quality that influence the AAS to a greater or lesser degree.^12^ For RCTs specifically, this information would be particularly useful when interpreting the AAS and would help delineate whether the AAS of such articles could be used as a proxy for well-constructed scientific methodology or whether it is being influenced by other factors. This is imperative to understand because RCTs often generate findings that directly affect and change clinical practices.^13,14^

Despite emerging research on AAS and the widespread use of AAS in orthopaedic surgery and total joint arthroplasty (TJA) journals, the relationship between AAS and methodologic quality or bias remains poorly understood. Given the increasing emphasis on quality within orthopaedic surgery and findings that come from RCTs, it is imperative to determine whether such biases may contribute to the AAS and the attention that an article receives. Therefore, the purpose of the current study was to determine the relationship between methodologic quality and study biases and the AAS in RCTs published in TJA journals. The authors hypothesized that there would be no statistically significant relationship between the methodological quality or study biases of RCTs and the AAS within the TJA literature.

## Methods

### Journal and Article Selection

Institutional board approval was not required to perform the current study. A total of seven prominent journals that publish TJA literature were queried for all RCTs published in the year 2016. These journals included *The Bone and Joint Journal*, *The Journal of Bone and Joint Surgery*, *Clinical Orthopaedics and Related Research*, *The Journal of Arthroplasty*, *The Journal of Knee Surgery*, *Acta Orthopaedica*, and *Hip International*. The year 2016 specifically was chosen as previous bibliometric studies have recommended a period of 3 to 4 years for analysis of citation rates and AAS to allow for an appropriate period of citation and AAS accumulation after publication.^9,15,16^ Furthermore, these seven journals were chosen specifically given that these journals represented those with the highest impact factors in the year 2016 and routinely published TJA articles.^17,18^ All RCTs were extracted from these journals, regardless of the subject and follow-up, because the primary aim of this study was to study the effect of methodologic and study bias on the AAS.

### Methodologic Quality Assessment

The primary outcomes of interest were the AAS, the methodologic bias of each study as quantified through the JADAD scale, and the type and number of study biases in each article. The JADAD Scale^19^ consists of a five-point questionnaire used to critically evaluate the methodologic quality of RCTs. The following questions are used to assess each study: (1) Was the study described as random; (2) Was the randomization scheme described and appropriate; (3) Was the study described as double blind; (4) Was the method of double blinding appropriate; and (5) Was there a description of dropouts and withdrawals? The scale is graded from 0 to 5 (a score of greater than or equal to 3 indicates a high-quality study, whereas a score less than 3 is considered to be low-quality).

### Study Bias Assessment

Study bias was assessed using the Cochrane risk of bias (RoB) tool for RCTs. This RoB tool was developed by Cochrane to introduce consistency and transparency in RCTs and has been subsequently validated. To make the process of assessing RoB more consistent and transparent, Cochrane developed and validated the Cochrane RoB tool.^20^ The most recent version of the Cochrane RoB tool,^21^ which was used in the current study, consists of six types of potential study biases which are classified in one of three ways: low RoB, high RoB, and unclear RoB. The six potential types of biases and the method by which they are assessed are shown in Table 1 and include (1) selection bias, (2) performance bias, (3) detection bias, (4) attrition bias, (5) reporting bias, and (6) other bias. For the current study, we considered high RoB to represent bias that was present in a study. If the RoB was low or unclear, the study was not documented as being influenced by that particular bias.

**Table 1:** Cochrane Risk of Bias Tool and Descriptions

Bias Domain	Bias Source	Description	High Risk	Low Risk	Unclear Risk
Attrition	Incomplete outcome data	Described the completeness of outcome data for each main outcome, including attrition and exclusions from the analysis. Stated whether attrition and exclusions were reported, the numbers in each intervention group (compared with total randomized participants), reasons for attrition/exclusions where reported.	Attrition bias because of amount, nature or handling of incomplete outcome data	Handling of incomplete outcome data was complete and unlikely to have produced bias	Insufficient reporting of attrition/exclusions to permit judgment of “low risk” or “high risk” (eg, number randomized not stated, no reasons for missing data provided)
Detection	Blinding of outcome assessment	Described all measures used, if any, to blind outcome assessors from knowledge of which intervention a participant received. Provided any information relating to whether the intended blinding was effective.	Detection bias because of knowledge of the allocated interventions by outcome assessors.	Blinding was likely effective	Not described in sufficient detail
Performance	Blinding of participants and personnel	Described all measures used, if any, to blind study participants and personnel from knowledge of which intervention a participant received. Provided any information relating to whether the intended blinding was effective	Performance bias because of knowledge of the allocated interventions by participants and personnel during the study.	Blinding was likely effective.	Not described in sufficient detail
Reporting	Selective reporting	Stated how the possibility of selective outcome reporting was examined by the authors and what was found.	Reporting bias because of selective outcome reporting.	Selective outcome reporting bias not detected	Insufficient information to permit judgment (it is likely that most studies will fall into this category)
Selection	Random sequence generation	Described the method used to generate the allocation sequence in sufficient detail to allow an assessment of whether it should produce comparable groups.	Selection bias (biased allocation to interventions) because of inadequate concealment of allocations before assignment.	Random sequence generation method should produce comparable groups	Not described in sufficient detail
	Allocation concealment	Described the method used to conceal the allocation sequence in sufficient detail to determine whether intervention allocations could have been foreseen in advance of, or during, enrollment.	Selection bias (biased allocation to interventions) because of inadequate concealment of allocations before assignment.	Intervention allocations likely could not have been foreseen in advance of, or during, enrollment	Not described in sufficient detail
Other	Any other bias, ideally prespecified	Any important concerns about bias not addressed above. If particular questions/entries were prespecified in the study's protocol, responses should be provided for each question/entry.	Bias because of complications not covered elsewhere in the table.	No other bias detected	There may be a risk of bias, but there is either insufficient information to assess whether an important risk of bias exists; or insufficient rationale or evidence that an identified complications will introduce bias.

### Altmetric Attention Score

Altmetric provides analyses of activity on various platforms social media platforms, which include Twitter, Facebook, news outlets, online blogs, Mendeley, Wikipedia, and others.^22^ The AAS is calculated through weighted scores of social media attention that a given published article receives and is updated in real-time.^23^ The score is subsequently quantified through an automated algorithm created by Altmetric. Given the dynamic nature of the AAS, all scores from the included RCTs were collected in a span of two days using the Altmetric Bookmarklet.^24^ The number of citations for each study was extracted from the Dimensions citation database, which is a platform affiliated with Altmetric. Dimensions reports the total number of times a work is cited and has been used in previous literature and deemed appropriate for collection of article citations.^25^

### Secondary Outcomes

In addition to the primary outcomes, predetermined bibliometric and social media-related variables were extracted for each RCT in accordance with previously published altmetrics studies.^9^ These variables included (1) the highest degree of first author, (2) total number of authors, (3) geographic region of origin of the publication, (4) disclosure of any conflict of interest (the presence or absence of general self-reported conflict of interest), (5) number of academic institutions, (6) involved joint (hip, knee, or both), (7) study topic, (8) study design, (9) sample size, (10) number of referenced studies, (11) number of Twitter mentions, (12) number of Facebook mentions, (13) number mentions by news outlets, (14) number of times referenced on Wikipedia, and (15) number of reads on Mendeley. These specific variables were chosen based on of factors found to be associated with citation rate and the AAS in previous literature.^26^

### Statistical Analysis

Normality was determined with the Shapiro-Wilks test, and subsequently continuous variables were presented as means with SDs or ranges where appropriate, and categorical outcomes were presented as frequencies with percentages. One-way analysis of variance with Bonferroni corrections for multiple comparisons or chi-squared tests of association were performed to compare bibliometric and Altmetrics characteristics, as well as AAS and citation rates, among journals. Univariate analysis with Pearson correlation coefficients and linear regression analysis was performed to determine the influence of methodologic and study biases on the AAS and citation rates. Multivariate linear regression was used to determine predictors of the AAS and citation rates for all RCTs. All statistical analyses were performed with Stata version 16.1 (StataCorp, College Station, TX). Statistical significance was defined as *P* < 0.05.

## Results

### Bibliometric Characteristics of Included Articles

A total of 42 RCTs were identified in the year 2016 among the seven journals. Most RCTs were published in *The Journal of Arthroplasty* (n = 9, 21.4%), followed by *Clinical Orthopaedics and Related Research* (n = 8), *The Journal of Bone and Joint Surgery* (n = 7), *The Bone and Joint Journal* (n = 7), *Acta Orthopaedica* (n = 6), *Hip International* (n = 3), and *The Journal of Knee Surgery* (n = 2). The mean (±SD) citations and AAS per RCT were 17.8 ± 16.5 (range, 0 to 78) and 8.0 ± 15.4 (range, 0 to 64), respectively. Additional baseline bibliometric characteristics are summarized in Table 2.

**Table 2:** Baseline Study Characteristics Including Bibliometric and Altmetric Variables

Characteristic	Journal							*P* Value
	BJJ (n = 7)	JBJS (n = 7)	CORR (n = 8)	JOA (n = 9)	JKS (n = 2)	Hip Int. (n = 3)	Acta (n = 6)	
Author degree								0.53
MD/DO	1 (14.3)	4 (57.1)	4 (50)	4 (44.4)	2 (100)	3 (100)	2 (33.3)	
Other								
Continent								**0.001**
North America	1 (14.3)	0 (0)	5 (62.5)	2 (22.2)	2 (100)	0 (0)	0 (0)	
Europe	4 (57.1)	5 (71.4)	2 (25)	2 (22.2)	0 (0)	0 (0)	6 (100)	
Asia	1 (14.3)	1 (14.3)	1 (12.5)	2 (22.2)	0 (0)	3 (100)	0 (0)	
South America	0 (0)	0 (0)	0 (0)	0 (0)	0 (0)	0 (0)	0 (0)	
Africa	0 (0)	0 (0)	0 (0)	0 (0)	0 (0)	0 (0)	0 (0)	
Australia	1 (14.3)	1 (14.3)	0 (0)	3 (33.3)	0 (0)	0 (0)	0 (0)	
COI								**0.005**
Yes	5 (71.4)	1 (14.3)	5 (62.5)	2 (22.2)	2 (100)	3 (100)	6 (100)	
No/not stated								
Joint studied								0.29
Hip	1 (14.3)	3 (42.9)	2 (25)	5 (55.6)	0 (0)	3 (100)	3 (50)	
Knee	6 (85.7)	4 (57.1)	6 (75)	3 (33.3)	2 (100)	0 (0)	3 (50)	
Both	0 (0)	0 (0)	0 (0)	1 (11.1)	0 (0)	0 (0)	0 (0)	
Arthroplasty								**<0.001**
Primary	6 (85.7)	5 (71.4)	7 (87.5)	7 (77.8)	0 (0)	3 (100)	6 (100)	
Revision	1 (14.3)	1 (14.3)	0 (0)	2 (22.2)	0 (0)	0 (0)	0 (0)	
Hemiarthroplasty	0 (0)	0 (0)	0 (0)	0 (0)	0 (0)	0 (0)	0 (0)	
Resurfacing	0 (0)	0 (0)	0 (0)	0 (0)	0 (0)	0 (0)	0 (0)	
Infection (one or two-stage exchange)	0 (0)	0 (0)	0 (0)	0 (0)	0 (0)	0 (0)	0 (0)	
Mega-prosthesis (non-oncologic)	0 (0)	0 (0)	0 (0)	0 (0)	0 (0)	0 (0)	0 (0)	
Unicompartmental	0 (0)	1 (14.3)	1 (12.5)	0 (0)	0 (0)	0 (0)	0 (0)	
Multiple/other	0 (0)	0 (0)	0 (0)	0 (0)	2 (100)	0 (0)	0 (0)	
Topic								**0.002**
Clinical/survivorship	5 (71.4)	5 (71.4)	6 (75)	8 (88.9)	1 (50)	3 (100)	2 (33.3)	
Imaging-based	2 (28.6)	2 (28.6)	0 (0)	0 (0)	0 (0)	0 (0)	0 (0)	
Preclinical	0 (0)	0 (0)	1 (12.5)	0 (0)	0 (0)	0 (0)	0 (0)	
Implant design	0 (0)	0 (0)	0 (0)	1 (11.1)	0 (0)	0 (0)	4 (66.7)	
Cost-analysis/economics/health policy	0 (0)	0 (0)	1 (12.5)	0 (0)	0 (0)	0 (0)	0 (0)	
Epidemiology	0 (0)	0 (0)	0 (0)	0 (0)	0 (0)	0 (0)	0 (0)	
Measure development/validation	0 (0)	0 (0)	0 (0)	0 (0)	0 (0)	0 (0)	0 (0)	
Kinematics/gait	0 (0)	0 (0)	0 (0)	0 (0)	1 (50)	0 (0)	0 (0)	
Other	0 (0)	0 (0)	0 (0)	0 (0)	0 (0)	0 (0)	0 (0)	
No. of authors	6.3 ± 1.8	4.9 ± 1.6	5 ± 1.9	5.9 ± 1.3	6.5 ± 0.7	6.0 ± 1.0	5.3 ± 1.8	0.53
No. of institutions	2.4 ± 1.4	1.4 ± 0.8	1.6 ± 1.0	1.7 ± 0.87	3.5 ± 0.7	1.0 ± 0.0	2.3 ± 1.2	0.08
No. of references	33.9 ± 6.0	31.0 ± 7.9	33.1 ± 15.2	25 ± 6.3	18 ± 4.2	26.7 ± 4.5	28.2 ± 6.5	0.20
Sample size	118.0 ± 71.5	84.7 ± 45.6	142.1 ± 163.6	178.3 ± 320.1	53 ± 24.0	124.0 ± 76.9	48.8 ± 16.4	0.84
Citations	17.9 ± 12.8	38.1 ± 29.6	19.3 ± 16.3	13.7 ± 12.3	6.5 ± 0.7	7.7 ± 11.6	6.8 ± 3.5	**0.033**
AAS	19.6 ± 25.4	13.3 ± 22.5	7.5 ± 3.6	4.6 ± 10.3	1.5 ± 0.7	0.7 ± 1.2	0.2 ± 0.4	0.25
Twitter mentions	7 (100)	5 (71.4)	8 (100)	7 (77.8)	2 (100)	1 (33.3)	1 (16.7)	**0.005**
Facebook mentions	7 (100)	2 (28.6)	8 (100)	0 (0)	1 (50)	1 (33.3)	0 (0)	**<0.001**
News outlet mentions	0 (0)	1 (14.3)	0 (0)	1 (11.1)	0 (0)	0 (0)	0 (0)	0.74
Blog mentions	0 (0)	4 (57.1)	0 (0)	1 (11.1)	0 (0)	0 (0)	0 (0)	**0.009**
Mendeley reads	45.9 ± 18.2	57.1 ± 20.0	69.4 ± 28.3	40.1 ± 14.5	56.5 ± 3.5	9.3 ± 16.2	16.2 ± 39.6	**0.003**
Wikipedia mentions	0 (0)	0 (0)	0 (0)	0 (0)	0 (0)	0 (0)	0 (0)	—

AAS = Altmetric Attention Score, Acta = *Acta Orthopaedica*, BJJ = *The Bone and Joint Journal*, CORR = *Clinical Orthopaedics and Related Research*, Hip Int. = *Hip International*, JBJS = *The Journal of Bone and Joint Surgery*, JKS = *The Journal of Knee Surgery*, JOA = *The Journal of Arthroplasty*

Statistics reported as frequencies (percentages) or mean ± SD. Bolded *P* values indicated statistical significance at *P* < 0.05 level.

### Influence of Methodological Bias on Altmetrics Attention Score and Citation Rate

**Figure 1:**
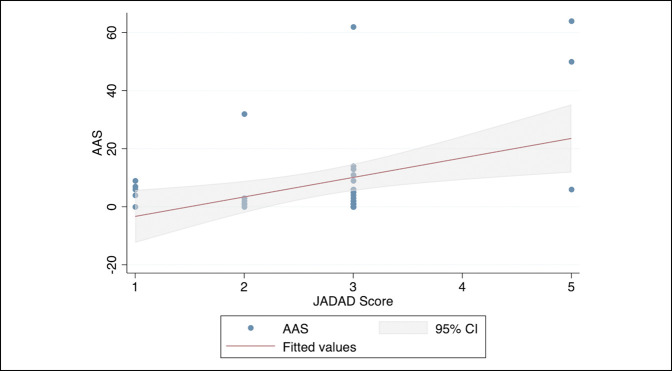
Linear regression model demonstrating relationship between the JADAD methodological quality score for RCTs versus the AAS for RCTs in seven total joint arthroplasty journals. AAS = Altmetric Attention Score, CI = confidence interval, RCT = randomized controlled trial

The mean JADAD score among all 42 RCTs was 2.6 ± 0.94. No statistically significant differences were observed in the JADAD score when compared across the seven journals (*P* = 0.57). Therefore, the journal variable was not controlled for in regression analysis. The linear regression model (Figure 1) was statistically significant (R^2^ = 0.17, *P* < 0.001) and demonstrated that higher JADAD scores were significantly and positively associated with higher AAS scores (β = 6.7, 95% confidence interval [CI], 2.0 to 11.4; *P* = 0.006). No significant association was found between citation rate and methodologic bias (*P* = 0.16).

### Influence of Study Bias on Altmetrics Attention Score and Citation Rate

**Figure 2:**
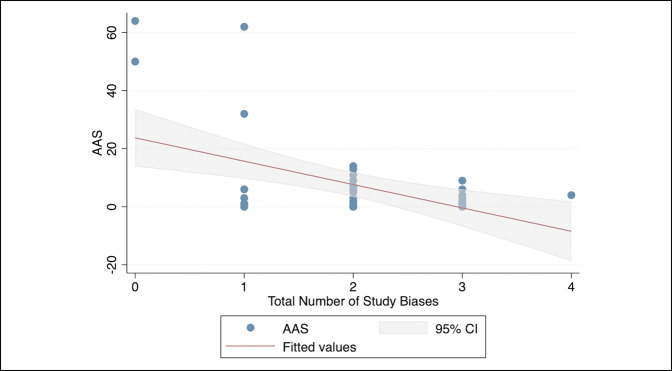
Linear regression model demonstrating relationship between the total number of study biases for RCTs versus the AAS for RCTs in seven total joint arthroplasty journals. AAS = Altmetric Attention Score, CI = confidence interval, RCT = randomized controlled trial

The mean total number of study biases among all 42 RCTs was 2.0 ± 0.93 (range, 0 to 4). No statistically significant differences were observed in the mean number of total study biases when compared across the seven journals (*P* = 0.27). Therefore, the journal variable was not controlled for in regression analysis. The linear regression model (Figure 2) was statistically significant (R^2^ = 0.24, *P* = 0.001) and demonstrated that a greater total number of study biases was significantly and negatively associated with higher AAS scores (β = −8.0, 95% CI, −12.6 to −3.5; *P* < 0.001). No significant association was found between total number of study biases and citation rate (*P* = 0.10).

The most frequent type of study bias found among the 42 RCTs was performance bias, with 39 (92.9%) studies demonstrating this bias. A high proportion of RCTs also demonstrated detection bias (n = 20, 47.6%) and attrition bias (n = 18, 43.9%), whereas only five studies (11.9%) demonstrated selection bias. No studies demonstrated reporting bias, although it was rated as an “unclear risk” in seven (16.7%) studies. Pearson correlation analysis demonstrated that performance bias had the strongest association with the AAS (r = −0.58, *P* = 0.001) and in regression was significantly and negatively correlated with the AAS (β = −34.4, 95% CI, −49.8 to −19.1; *P* < 0.001).

### Association Between Citation Rate and Altmetrics Attention Score in Total Joint Arthroplasty Randomized Controlled Trials

**Figure 3:**
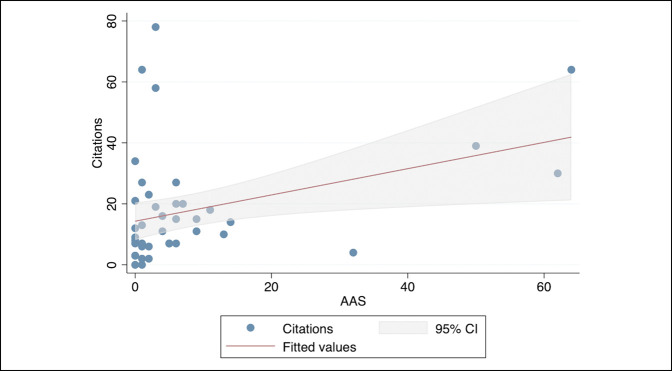
Linear regression model demonstrating relationship between AAS RCTs versus the citation rate for RCTs in seven total joint arthroplasty journals. AAS = Altmetric Attention Score, CI = confidence interval, RCT = randomized controlled trial.

Pearson correlation analysis demonstrated a significant and positive association between the AAS of articles and the number of citations (r = 0.36, *P* = 0.019), whereas no publication characteristics demonstrated statistically significant relationships. Subsequently, a linear regression model was created to model the relationship on a scalar response (Figure 3). This model demonstrated the higher AAS were significantly and positively associated with the citation rate of an RCT (β = 0.43, 95% CI, 0.073 to 0.79; *P* = 0.019).

### Predictors of Social Media Attention in Total Joint Arthroplasty Randomized Controlled Trials

All 11 bibliometric characteristics were tested for the magnitude of correlation with a study being mentioned on Twitter, Facebook, news outlets, blogs, Mendeley, and Wikipedia. No statistically significant associations were found between bibliometric characteristics and a RCT being mentioned on one of the aforementioned social media platforms.

## Discussion

The main findings of the current study were (1) higher JADAD scores were markedly and positively associated with higher AAS, indicating that the AAS is reflective of excellent methodologic quality in RCTs in the TJA literature; (2) fewer study biases were markedly and positively associated with higher AAS in RCTs in the TJA literature, suggesting that the AAS is reflective of the findings that are not influenced by bias; (3) AAS was markedly associated with citation rates for RCTs published in journals that routinely publish TJA research.

The JADAD score was markedly associated with the AAS, with higher JADAD scores being markedly and positively predictive of higher AAS. This finding suggests that more sound methodologic quality leads to higher AAS and social media attention for RCTs pertaining to TJA. Methodologic quality assessments of RCTs are imperative to avoid the incorporation of low-quality or potentially misleading recommendations into clinical practice.^27^ A large empirical study based on F1000Prime data recently suggested that Altmetrics was related to the quality of articles as evaluated by the postpublication peer-review system of F1000Prime assessments.^28^ Because Altmetrics continues to gain popularity among funding bodies, researchers cause wider research impact ranging from social media to production of policy documents, the current study substantiates the legitimacy of these roles by demonstrating that it reflects studies with reliable methodologic quality. TJA surgeons, researchers, and other individuals who choose to explore research based on Altmetrics can be confident in the findings presented by these RCTs because the AAS represents studies with high methodologic quality. However, we still recommend the critical appraisal of RCTs regardless of presumed quality before adoption of particular findings that may change clinical practice.

RCTs pertaining to hip and knee arthroplasty that were not subjected to inherent study biases such as attrition and performance bias were found to accrue more interest on social media platforms and had higher AAS. Interestingly, performance bias was the most frequent type of study bias, with the results of 92.9% of RCTs being influenced by this bias. Study biases may also negatively influence the results and should be considered in the interpretation of study results.^29-31^ This is especially true when concerning RCTs because these often generate practice-altering findings.^13,14,32^ Interestingly, articles with low AAS tended to have a greater number of total study biases, although the average number of total biases per RCT was low. Given that Altmetrics represents high methodologic quality and low study biases, we recommend the use of Altmetrics as both a screening tool for high-quality articles and as a measure of scientific quality for RCTs in the TJA literature.

Citation rate was markedly and positively influenced by the AAS, demonstrating that RCTs in the TJA literature that had high AAS also had greater academic impact. This finding is in accordance with previous Altmetrics-based studies in the literature. Kunze et al^9^ investigated the relationship between the AAS and citation rate of articles from five different orthopaedic sport medicine journals and found that a greater AAS score significantly predicted a greater citation rate (β = 0.16; *P* < 0.001). The current study determined a stronger relationship between the AAS and citation rate (β = 0.43, *P* = 0.019), suggesting that for every one-point increase in the AAS of an RCT in the TJA literature, approximately 0.5 citations will be gained accordingly. This finding has notable implications for journals, authors, and funding bodies because it suggests that the promotion of high-quality RCTs is associated with greater academic impact and ultimately more article citations.

This study has several limitations. First, only RCTs were included, and the relationship between AAS and methodologic and study bias may not be generalizable to studies with lower levels of evidence. However, the study of RCTs was a specific purpose of the current study because they designed to represent the highest quality evidence and often generate findings that change clinical practice. Second, we limited the current analysis to the use of only two quality appraisal tools—the JADAD scale and Cochrane RoB tools for RCTs. Although these are both validated tools that are routinely used in the orthopaedic literature, relationships between quality and AAS may not be generalizable with the use of other tools. Third, the current analysis represents that of a single year of RCTs, although previous Altmetrics studies have demonstrated that analyses from a single year are appropriate.^9^ Finally, Altmetrics does not currently disclose information related to self-promotion by authors and journals, and the current study could not control for this or for random article clicks and shares.

## Conclusion

High methodologic quality and limited study bias markedly contribute to the AAS of RCTs in the TJA literature. The AAS may be used as a proxy measure of scientific quality for RCTs, although readers should still critically appraise these articles before making changes to clinical practice.
